# The Virtualization of a Movement and Social Group-Activity Intervention for Older Adults and Their Caregivers

**DOI:** 10.1093/geroni/igab046.1090

**Published:** 2021-12-17

**Authors:** Christina Hugenschmidt, Deepthi Thumuluri, Christina Soriano, Rebecca Barnstaple, Jason Fanning, Jessie Laurita-Spanglet, Edward Ip

**Affiliations:** 1 Wake Forest School of Medicine, Winston-Salem, North Carolina, United States; 2 Wake Forest School of Medicine, winston-Salem, North Carolina, United States; 3 Wake Forest University, Winston-Salem, North Carolina, United States; 4 York University, Toronto, Ontario, Canada; 5 Wake Forest University, Winston Salem, North Carolina, United States; 6 University of Southern Maine, Portland, Maine, United States

## Abstract

COVID-related safety concerns mandated suspension of our ongoing trial testing the effects of movement and social engagement in older adults with early-stage dementia and their caregivers (dyads). Participant vulnerability and the requirement for group social interaction complicated intervention resumption. We present results from a successful pilot to rapidly and iteratively optimize study interventions for remote delivery targeting intervention mediators (social connection, movement) based on participant feedback. Three-dyad groups (n=6 individuals) completed cycles of intervention via Zoom immediately followed by an interview with open-ended and quantitative feedback. Cycles were repeated until no new information was solicited, then repeated with new participants. Optimization revealed needs for technological support, more intensive movement, and social connection. Specifically, the inability to make eye contact, see others’ full body, and technology-associated timing asynchronies impeded social connection in the movement group. We will present practical tips for crafting remote group interventions for caregiver/person living with dementia dyads.

